# Evaluations of guided bone regeneration in canine radius segmental defects using autologous periosteum combined with fascia lata under stable external fixation

**DOI:** 10.1007/s10195-014-0321-z

**Published:** 2014-10-12

**Authors:** Zhe Yu, Jie Geng, Haoran Gao, Xinwen Zhao, Jingyuan Chen

**Affiliations:** 1 Department of Orthopedic Surgery, Tangdu Hospital, Fourth Military Medical University, Xi’an, 710038 Shaanxi People’s Republic of China; 2 Medical Department of Tangdu Hospital, Fourth Military Medical University, Xi’an, 710038 Shaanxi People’s Republic of China; 3 Faculty of Military Preventive Medicine, Fourth Military Medical University, 169 Changle West Road, Xi’an, 710032 Shaanxi People’s Republic of China

**Keywords:** Guided bone regeneration, Periosteum, Bone defect, Bone formation

## Abstract

**Background:**

Although bone defect is one of the most common orthopaedic diseases, treatment remains a challenge and an issue of debate. Guided bone regeneration (GBR) is primarily accompanied by barrier membranes; however, optional membranes show some inherent flaws in clinical application. The purpose of this study was to observe the healing velocity and quality of repairing canine radius segmental defect using transferred autologous periosteum combined with fascia lata, which can provide better biological safety than other materials.

**Materials and methods:**

Twenty adult male beagles weighing 11.45 ± 1.29 kg were used as animal models. The animals were randomly allocated into three groups, a blank control group, a fascia lata control group and a combined fascia lata and periosteum group. Standardised artificial bony defects were prepared at the radius and treated with autologous periosteum combined with fascia lata under stable external fixation. The newly formed bone-growth curve was made according to ultrasound (US) detection, and histopathologic and scanning electronic microscope (SEM) evaluations were also performed.

**Results:**

Bone union was seen in most individuals from the autologous periosteum combined with fascia lata group, within an average of 14.2 weeks. Histopathologic and SEM examinations both showed the different osteogenesis state between groups. Necropsy confirmed US findings with regard to distance of bone defects and location.

**Conclusion:**

These findings suggest that autologous periosteum combined with fascia lata is as effective as a GBR membrane, even in long tubular bone defects. With reliable biological safety, the autologous periosteum combined with fascia lata is expected to achieve increasing application in orthopaedic trauma patients.

**Level of evidence:**

Not applicable, animal study.

## Introduction

Bone healing is one of the most important processes in the orthopaedic clinical field, especially following osteomyelitis, nonunion, tumours and plastic surgery. In general, a major obstacle to bone healing and formation of new bone is the rapid formation of connective tissue, which prevents osteogenesis [1]. Since Gottlow et al. [2] successfully cured periodontal diseases by using the barrier membrane technique in 1982, guided tissue regeneration (GTR) [3, 4] and guided bone regeneration (GBR) [5, 6] have been successively applied in clinical settings. Both GTR and GBR use a barrier membrane to prevent epithelial migration and the appearance of connective tissue, and GBR also aims to promote bone regeneration. For more than a decade, this technique has been applied in clinical dentistry for various purposes, including dental implant therapy with an insufficient bone volume in the recipient site [7–9].

Since the GBR technique depends primarily on the use of barrier membranes, these membranes and their properties play an important role in outcomes. At present, nonbioresorbable and bioresorbable membranes are the two main types of barrier membranes available [10]. Expanded polytetrafluoroethylene (e-PTFE) is the most commonly used nonbioresorbable membrane [11], while collagen membrane is the most commonly used bioresorbable membrane [12]. However, neither is ideal for use in GBR. Although the e-PTFE membrane has been confirmed to have excellent biocompatibility in many studies, it requires a second surgical procedure for its removal because of its nonresorbability. On the other hand, the collagen membrane is resorbable, but it has inherent disadvantages, such as poor structural integrity, variable degradation rates and host immune reactivity [13]. Thus, in order to promote effective bone regeneration using the GBR approach, the barrier membrane used must have specific properties in terms of bioactivity (osteoconductivity) and bioresorption, as well as space-maintaining ability, which is related to its mechanical stability.

Periosteum can meet some prerequisites for tissue-engineered bone repair, as it contains pluripotential mesenchymal stem cells with the potential to form either cartilage or bone [14]. Periosteum has two discrete layers: an outer fibrous layer and an inner cambial layer. The fibrous layer appears to be composed of fibroblastic cells in a collagen and elastin fibre matrix, along with a nerve and microvascular networks. The cambium layer is highly cellular and contains numerous cell types, including fibroblasts, osteoblasts, and osteochondral precursor cells. Mesenchymal precursor cells in the periosteum differentiate into neochondrocytes, producing cartilage tissue during embryogenesis and contributing to bone apposition during intramembranous ossification by differentiating into osteoblasts [15]. Because it can be transplanted as a whole tissue, it can serve as its own scaffold or a matrix onto which other cells and/or growth factors can adhere. To further ensure the space-maintaining ability, we selected the fascia lata in order to increase supporting strength. This tissue is adjacent to the autologous periosteum donor organ of the femur and can provide considerable supporting effects that other soft tissue cannot.

According to the abovementioned principles, we proposed the use of autologous periosteum combined with fascia lata using a stable external fixation frame as a barrier membrane. The advantages of this membrane are that host immune reactivity need not be taken into consideration, and no surgery is required for its removal. To our knowledge, the effect of using autologous periosteum or fascia lata has not been examined previously in detail. Therefore, this study aimed to investigate local changes in new bone formation following bone defects in canine radii treated by the GBR technique with autologous periosteum combined with fascia lata under stable external fixation. We also wanted to identify local events occurring in response to the periosteum using imaging, histological examination, and scanning electron microscopy (SEM).

## Materials and methods

All experimental procedures involving animals were conducted using a protocol reviewed and approved by the Ethics Committee of Tangdu Hospital, Fourth Military Medical University (Permit Number: 2012028). All work was carried out in accordance with national and international guidelines to minimise animal suffering. Adult male beagle dogs were purchased from the Laboratory Animal Research Centre of the Fourth Military Medical University of China. Animal experiments were conducted at the Orthopedics Oncology Institute of Chinese PLA. The dogs were housed in microisolator cages under specific pathogen-free conditions. The unilateral external fixation frame used in this study is commercially available. It is designed by Xia Hetao and is commonly used for upper-limb fractures in humans [16].

**Fig. 1:**
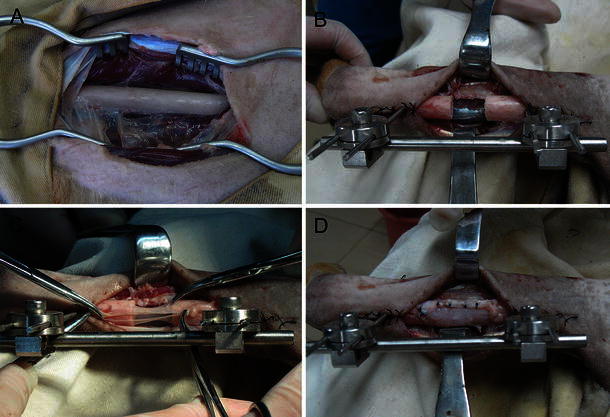
Procedural steps: **a** exposure of canine radius; **b** unilateral external fixation and creation of a bone defect in the radius; **c** transplantation with autologous periosteum to preserve the osteogenic area; **d** suture fixation of the dissociated fascia lata

Twenty adult male beagle dogs weighing 11.45 ± 1.29 kg were randomly divided into three groups: A (*n* = 6), the blank control group, in which bone defects were fixed with a unilateral external frame and left to heal spontaneously; B (*n* = 6), the fascia lata control group, in which the externally fixed bone defects were covered and sutured with autologous fascia lata, without the periosteum; and C (*n* = 8), the periosteum combined with fascia lata group, in which the externally fixed bone defects were covered with autologous fascia lata to preserve the potential osteogenic area, following which the fascia lata was fixed with sutures to ensure adequate support and isolation. Surgical procedures were performed under aseptic conditions by the same surgical team and with the animals under general anaesthesia induced using isoflurane gas, in conjunction with endotracheal intubation. The experimental outline is illustrated in Fig. 1.

At the beginning of the experiment, a 15-mm defect was surgically created in a single radius (Fig. 1a, b). Simultaneously, the autologous periosteum and fascia lata were obtained from the left proximal femur and prepared for further transplantation. Unilateral external fixation was conducted according to biomechanical and humanitarian principles while minimising the time of the surgery to in turn minimise the animals’ suffering.

In group C, the prepared autologous periosteum was sutured into the living periosteum at the fixed bone end of osteotomy to preserve the potential osteogenic area (Fig. 1c). To obtain adequate support and isolation, the dissociated fascia lata was fixed with sutures (Fig. 1d). In group B, the prepared fascia lata was transplanted into the segmental defects alone. In group A, the defects were left to heal spontaneously.

All incisions were closed using interrupted silk sutures. At this point, the process of surgical interference was complete. Postoperatively, the animals were administered 1 g of amoxicillin once daily for 5 days. Sutures were removed 14 days after surgery, and the animals were fed a soft laboratory diet for the study duration. Further, the operated areas and general conditions of the animals were checked daily according to standard veterinary postoperative care.

On a monthly basis, X-ray examination was conducted in live animals to study newly formed bone. Images were acquired such that they included the adjacent elbow and wrist joints. An X-ray microtomography (Micro CT SkyScan 1072; SkyScan, Kontich, Belgium) was used for this purpose, without any preparation. For ultrasound (US) imaging, a 5.0-MHz real-time scanner (SSA-550; Toshiba Medical Systems, Tokyo, Japan) was used. This procedure was conducted weekly, and a Doppler digital image optimiser (Toshiba Medical Systems, Tokyo, Japan) was used, which enables adaptive image processing for high sensitivity. The standard for bone healing was defined as disappearance of the defect area on US images, and then new bone growth curve was created.

After 20 weeks, the animals were sacrificed with an overdose of thiopental sodium. The radii were removed, block-resected using an oscillating saw and prepared for histological examination. The 20 bone blocks were immersed in a solution of 4 % formaldehyde, dehydrated in ethanol, and embedded in methyl methacrylate. Nondecalcified sections of ~300-mm thickness were obtained using a low-speed diamond saw with coolant. The sections were glued onto opalescent acrylic glass, ground to a final thickness of ~80 mm and surface stained with toluidine blue and basic fuchsin. To observe the morphology of the newly formed bone, sections were fixed with 1.5 % glutaraldehyde in 0.1 M phosphate-buffered saline (pH 7.4), passed through an alcohol gradient, dried in a Ladd Critical Point Dryer and coated with platinum in a Polaron SEM coating system. The fixed sections were examined with a JEOL JSM-35CF SEM, and SEM studies were performed with backscattered electrons at 15 kV in conjunction with image analysis. The quantity of newly formed bone between the two ends of the osteotomy was analysed.

All samples were dehydrated in graded ethanol and acetone. Nondecalcified bone specimens were infiltrated and embedded in glycolmethacrylate resin. For each sample, 7-μm serial sections were cut perpendicular to the newly formed bone using a diamond saw (Reichert-Jung Supercut 2050) and fixed in buffered isotonic formaldehyde (100 ml 37 % formaldehyde solution, 900 ml distilled water, 4 g monobasic sodium phosphate, 6.5 g dibasic sodium phosphate) and embedded in paraffin. After 24 h, samples were immersed in 70 % alcohol, stained with hematoxylin–eosin (H&E) and examined histopathologically by a blinded pathologist using a light microscope (Leica DM-RBE microscope) equipped with a high-resolution video camera (Q-500 MC; Leica) coupled to a computer monitor. SPSS software (version 11.0; SPSS, Chicago, IL, USA) was used for data variation analysis. The length of the defect area measured from X-ray and ultrasound images and expressed as average ± standard error (SE) was compared among groups using Student’s *t* test if the variables adjusted to a Gaussian distribution, with statistical significance set at *P* < 0.05. Means were compared using Kruskal–Wallis tests if data did not follow normal distribution. Bone healing rates were determined using the χ^2^ test, for which *P* < 0.05 was again considered to indicate statistical significance.

## Results

**Fig. 2:**
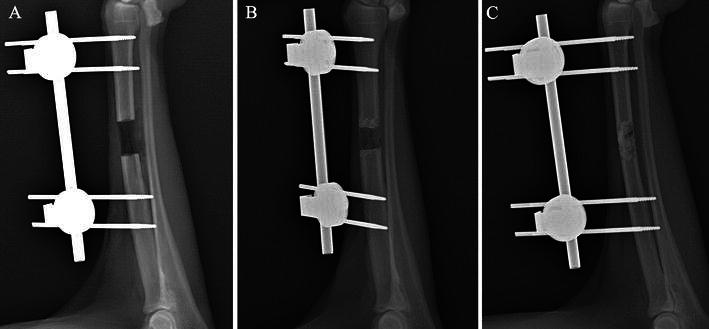
X-ray comparison of union and nonunion: **a** group A showed minimal bone callus coverage and changes in defect lengths; **b** most bone defects in group B had a similar bone healing rate as group A, showing callus abundance and reduced bone defect size as changes; **c** complete bone healing in group C shows newly formed bone rebuilt in accordance with the original radius

X-ray examination showed that in the blank control group, there was minimal proliferation at the end of the osteotomy surface immediately after the operation, and even 20 weeks later, bone defects showed minimal bone callus coverage with hardly distinguishable changes in length (Fig. 2a). Additionally, US images indicated that the distance between the two osteotomy ends also showed little change, and the final length of the defect area was 12.4 ± 2.43 mm (Table 1).

**Table 1:** Bone healing rate and average bone defects

Groups	Intervention means	Animal numbers	Average bone defects (mm)	Average union time (weeks)	Animals with bone healing	Bone healing rate (%)
Group A	Blank control group	6	12.4 ± 2.43	0	0	0
Group B	Fascia lata control group	6	7.58 ± 5.38	17	1	16.7
Group C	Periosteum combined with fascia lata group	8	1.63 ± 3.2	14.2 ± 2.75	6	75

In the fascia lata control group, bone growth varied substantially among individual animals. Most bone defects showed a similar healing rate as the blank control group, showing callus abundance and reduced bone defect size (Fig. 2b). Only one animal showed rough bone union, in the 17th postoperative week, with proof of defect area disappearance on US imaging. The final length of the defect area in this group was 7.58 ± 3.74 mm.

In the periosteum combined with fascia lata group, bone union was observed in most individuals after an average period of 14.2 weeks (Fig. 2c). In fact, the newly formed bone was rebuilt in accordance with the original radius. Only two animals showed apparent bone defects on US imaging throughout the monitoring period. The final length of the defect area was 1.63 ± 3.2 mm.

US imaging showed persistent bone defect gaps in all animals in group A. In group B, only one animal showed a gradually reducing low-echo mass on uUS images. Then, rough bone union was observed after 17 weeks, and the low-echo area disappeared. Of the remaining five animals in group B, neither apparent callus formation nor bone union was observed.

**Fig. 3:**
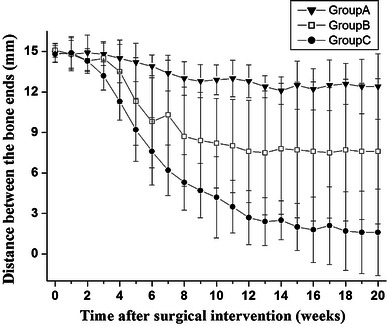
Time course of new bone growth. Length of bone defect measured and calculated weekly after surgical intervention. Data represent mean distance ± standard deviation

In general, 75 % of bone defects in group C healed within the 20-week study period; the median healing time was 14.2 ± 2.75 weeks (Fig. 3). The progress of bone healing was most evident during the 4–12 weeks after fracture. In two animals, bone defects were detected on US images throughout the monitoring period.

**Fig. 4:**
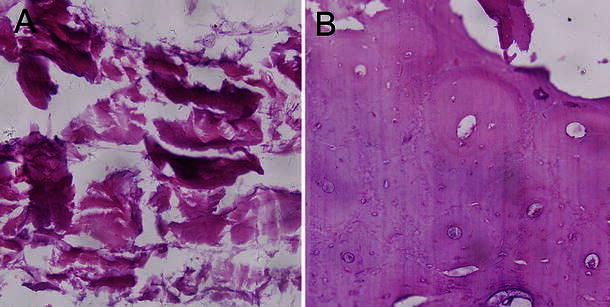
Histopathological examination of radii blocks using hematoxylin & eosin staining: **a** fibrous connective tissue was observed in the bone defect gaps in group A; **b** group C individuals with bone healing showed abundant cartilaginous callus and tightly packed trabecular bone

Histopathologic observation of the radii blocks showed that mostly fibrous connective tissue and a small amount of cartilaginous bone callus were present in the bone defect gaps in group A animals (Fig. 4a). However, in group C individuals showing bone healing, the presence of more abundant cartilaginous callus than the control group was histologically confirmed. Further, trabecular bone was tightly packed, and even the rebuilt Haversian canal system could be clearly distinguished (Fig. 4b). From one osteotomy end to the other, there was no visible defect gap in the newly formed bone area.

**Fig. 5:**
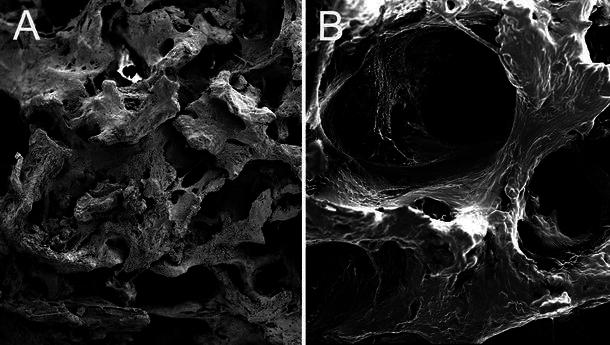
Scanning electron microscopy (SEM) of radii blocks: **a** fibrous connective and muscle tissues were seen in the defect gaps in group A; **b** spongy newly formed bone was seen in group C, and the bone-forming surface had osteoblastic lacunae

SEM showed that the newly formed bone was connected to both osteotomy ends of the host bone by cartilaginous bone callus. The newly formed bone had a spongy appearance with many vascular spaces, and the bone-forming surface had osteoblastic lacunae (Fig. 5b). However, bone-defect gaps in group A were mostly filled with fibrous connective tissue and muscle tissue (Fig. 5a), and no signs of bone healing were detected between osteotomy ends.

**Fig. 6:**
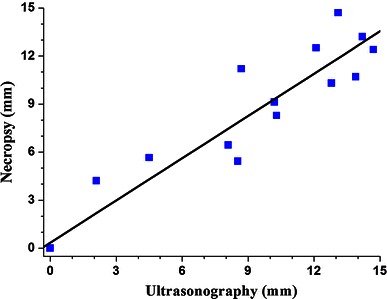
Simple linear regression test showing a positive correlation between ultrasound and necropsy findings regarding length of bone defects (mm) (*r*^2^ = 0.924; *P* < 0.05)

On the day they were sacrificed, all animals was examined by Doppler US to obtain final values of bone-defect gaps. The animals were then necropsied, and the radii blocks were removed using a sliding calliper and examined histologically. Data of all 20 dogs were compared. A simple linear regression test showed a positive correlation between bone-defect length (mm) detected using US and necropsy (*r*^2^ = 0.924; *P* < 0.05) (Fig. 6). Thus, necropsy confirmed US findings with regard to bone-defect length and location.

## Discussion

Although bone defects are a common problem following injury, bone tumour or other pathologies, treating this disease remains a challenge and an issue of debate. With regard to treatment, the Ilizarov technique occupies an important position. This technique involves applying a stable external fixator made from thin wires and brackets and performing osteotomy with a minimal incision [17]. When connecting struts are moved toward the bone defect, new soft bone will stretch and form between osteotomy ends as a result of the stretch-stress stimulus, which promotes bone formation from fibroblast- and periosteum-derived bone [18]. Among the many steps of the Ilizarov procedure, preserving local blood supply and considerable integrity of periosteum probably play the most critical roles in bone formation. The periosteum not only preserves local osteogenesis but also acts as a barrier membrane combined with the fascia lata to prevent soft tissue invasion. Connective tissue formation is a major obstacle to bone healing and new bone formation. The presence of connective tissue at the bone defect site prevents osteogenesis, resulting in incomplete healing or nonunion [19]. If an enclosed space is created by periosteum, many growth factors, such as bone morphogenetic protein, alkaline phosphatase, osteopontin, osteocalcin, could be preserved to induce newly formed bone. The molecular mechanism of periosteum GBR probably resulted from the combined effects of the several factors mentioned earlier. Therefore, we developed an autologous periosteum transfer strategy wherein the periosteum combined with the fascia lata is applied directly to the osteotomy ends of the bone defect under stable external fixation. Membrane placement promotes osseous healing in bone defects by excluding competing nonosteogenic soft tissue cells from the bone-defect site. In addition, the space enclosed by the periosteum and fascia lata protects haematopoietic stem cells and bone progenitor cells from leakage or dilution and can provide considerable osteoblast activity and inductive capability with the collection of variable growth factors. In general, the principle of this technique is an extension of the barrier membrane technique, wherein a space protected from competing connective tissue invasion is provided in the defect gap to promote osteogenesis.

Compared with nonbioresorbable and bioresorbable membranes, the autologous periosteum combined with the fascia lata is a more natural alternative. Although nonbioresorbable membranes have been successfully used in several situations [20, 21], they usually cannot remain long in the living body with confirmed biological safety. Further, a second surgical procedure is required to remove these membranes because of their natural nonresorbability. In contrast, bioresorbable membranes do not require a secondary surgery, but they have weak structural integrity and variable degradation rates and show host immune reactivity [22]. Autologous periosteum combined with the fascia lata, which can be easily obtained from the adjacent bone surface, can be used to overcome these drawbacks. This alternative membrane is not associated with the risks of degradation, host rejection or biological toxicity and requires no secondary surgery. Thus, autologous periosteum combined with the fascia lata seems to have the greatest biological safety.

A significant finding in this study was the variation in growth rates of newly formed bone and bone-healing velocity amongst groups. Standardised artificial bony defects were created in the canine radius and covered with the periosteum combined with the fascia lata. In the first 3 weeks, no animal showed evident osteogenesis activity and only minimal bone callus formation adjacent to the osteotomy ends on US. Bone healing was most evident 4–12 weeks after fracture. In group C, most animals showed a gradually reducing defect gap on US monitoring, although the final average defect length was high, at 1.63 ± 3.2 mm, because two animals showed evident bone defects 8.53 and 4.51 mm long. Necroscopic examination in a nonunion animal model showed that the anastomotic site of the periosteum and fascia lata failed to heal because of the presence of soft tissue at one osteotomy end, because of which the newly formed bone lost its union bridge. In group B, all anastomotic sites were confirmed for suture-assisted tissue healing.

Currently, the most commonly used technique for detecting bone defects and formation is X-ray examination. However, other techniques have also been used to characterise bone growth; for example, scintigraphy, microcomputed tomography-X, computed tomography and magnetic resonance imaging. Additionally, novel analytical tools are in development and may be adaptable to dogs. For instance, positron emission tomography can be performed with 2-[^18^F]-fluoro-2-deoxy-d-glucose or 99mTc-bisphosphonate to detect the level of osteogenesis. Although Doppler US is not an emergent method, it is still valued by researchers in this field. It is convenient, noninvasive and can be used to visualise and measure bone growth at any stage, including growth of primary bone callus and the assessment of angiogenesis in the pathological region, even in living organisms.

In conclusion, to our knowledge, this is the first study to evaluate the efficacy of GBR treatment of long-bone defects by directly applying autologous periosteum combined with the fascia lata to the osteotomy ends under stable external fixation. It is also the first report confirming that the GBR technique could be effective for treating long tubular bone defects, which remains a challenge for orthopaedic surgeons, and it may enable superior bony union across a considerable bone defect gap of almost 15 mm. In our future research, we will conduct detailed immunocytochemical assays in order to determine the molecular mechanisms underlying GBR after autologous periosteum transplantation. Because of its reliable biological safety, we believe that autologous periosteum combined with the fascia lata will be applied more commonly in the orthopaedic trauma field.

## Abbreviations

BMT: Barrier membrane technique
GBR: Guided bone regeneration
GTR: Guided tissue regeneration
SEM: Scanning electronic microscope
ALP: Alkaline phosphatase
OP: Osteopontin
OC: Osteocalcin
